# QuCo: quartet-based co-estimation of species trees and gene trees

**DOI:** 10.1093/bioinformatics/btac265

**Published:** 2022-06-27

**Authors:** Maryam Rabiee, Siavash Mirarab

**Affiliations:** Department of Computer Science and Engineering, University of California, San Diego, La Jolla, CA 92093, USA; Department of Electrical and Computer Engineering, University of California, San Diego, La Jolla, CA 92093, USA

## Abstract

**Motivation:**

Phylogenomics faces a dilemma: on the one hand, most accurate species and gene tree estimation methods are those that co-estimate them; on the other hand, these co-estimation methods do not scale to moderately large numbers of species. The summary-based methods, which first infer gene trees independently and then combine them, are much more scalable but are prone to gene tree estimation error, which is inevitable when inferring trees from limited-length data. Gene tree estimation error is not just random noise and can create biases such as long-branch attraction.

**Results:**

We introduce a scalable likelihood-based approach to co-estimation under the multi-species coalescent model. The method, called quartet co-estimation (QuCo), takes as input independently inferred distributions over gene trees and computes the most likely species tree topology and internal branch length for each quartet, marginalizing over gene tree topologies and ignoring branch lengths by making several simplifying assumptions. It then updates the gene tree posterior probabilities based on the species tree. The focus on gene tree topologies and the heuristic division to quartets enables fast likelihood calculations. We benchmark our method with extensive simulations for quartet trees in zones known to produce biased species trees and further with larger trees. We also run QuCo on a biological dataset of bees. Our results show better accuracy than the summary-based approach ASTRAL run on estimated gene trees.

**Availability and implementation:**

QuCo is available on https://github.com/maryamrabiee/quco.

**Supplementary information:**

Supplementary data are available at *Bioinformatics* online.

## 1 Introduction

Species tree estimation from multi-locus genome-wide datasets while accounting for gene tree discordance is now routinely attempted. There has been much effort to develop methods that can infer species trees and gene trees accurately, and in particular, methods focused on handling incomplete lineage sorting (ILS) as modeled by multi-species coalescent (MSC) (Rannala *et al.*, 2020) abound (e.g. Chifman and Kubatko, 2014; Liu, 2008; Liu *et al.*, 2010; Mirarab *et al.*, 2014a; Ogilvie *et al.*, 2017; Vachaspati and Warnow, 2015). Yet, a major challenge remains. The most accurate methods for species tree estimation have been Bayesian methods that co-estimate gene trees and species trees, while the most scalable methods have been summary methods that first estimate gene trees independently and then combine them to infer a species tree (Mirarab *et al.*, 2021). This dichotomy has left practitioners with a choice between using methods that can handle large data or more accurate methods that will have to be run on subsets of the data (Smith *et al.*, 2014). Neither solution is ideal.

Theoretical expectations and empirical evidence suggest that inferring all gene trees together should be more accurate than the two-step approach that independently computes gene trees (Bayzid and Warnow, 2013; Dasarathy *et al.*, 2015; Leaché and Rannala, 2011; Ogilvie *et al.*, 2016; Szöllősi *et al.*, 2015). Gene tree estimation from limited-length locus data is error-prone, and gene tree estimation error impacts species trees (Huang *et al.*, 2010; Lanier and Knowles, 2015; Mirarab *et al.*, 2014b; Molloy and Warnow, 2018; Patel, 2013). Importantly, gene tree estimation error is not just random noise and can create biases. Roch *et al.* (2019) have proved that under challenging cases, long-branch attraction in gene trees could lead to long-branch attraction in species trees and statistically inconsistent estimates. Dealing with gene tree estimation error has motivated several remedies, including binning genes (Bayzid and Warnow, 2013; Mirarab *et al.*, 2014b), collapsing low support branches (OneKP Initiative, 2019; Zhang *et al.*, 2018), and sampling posterior distributions of gene trees (Bossert *et al.*, 2021). However, these methods have their own drawbacks. Binning has the possibility of combining discordant genes, collapsing low support requires selecting a threshold, and simply combining samples from gene tree posteriors as input to summary methods shows mixed results in simulations (Mirarab, 2019). Thus, co-estimation methods are still the ideal option. Yet, current co-estimation methods such as *BEAST (Ogilvie *et al.*, 2017) scale only to tens of species (McCormack *et al.*, 2009).

The scalability of co-estimation methods has remained limited because they address a challenging problem using the slow MCMC process. The space of parameters for the joint species/gene tree inference is extremely large and consists of both discrete (gene tree and species tree topologies) and continuous parameters (per-branch lengths and rates, population sizes, and per-gene transition rate matrices). To explore this large and heterogeneous space to convergence, MCMC needs to run for a long time. Developing theoretically justified scalable co-estimation methods requires simplifying the model and heuristic methods. Wang and Nakhleh (2018) sped up co-estimation by avoiding full sampling of the entire space using an EM-like algorithm that iteratively switches between species tree and gene tree estimation. While this method increased gene tree accuracy, it was not clear that it improved species tree accuracy, perhaps because the estimated species tree was used to improve gene trees.

This article is an attempt at providing a likelihood-based approach to co-estimation under MSC designed to scale using simplifying assumptions and heuristics while keeping a likelihood-based core. We observe that while joint sampling of *continuous* parameters of gene trees, such as their branch lengths, slows down co-estimation, these are often nuisance parameters. Therefore, we focus on topology, marginalizing over gene tree branch lengths and other continuous parameters. However, this marginalization would still be intractable if done jointly. Instead, we ignore the dependency among substitution unit branch lengths (similar to a no-common-mechanism model) and assume continuous parameters across gene trees are fully unlinked. This admittedly strong assumption enables us to decouple genes. We first use existing methods to estimate gene tree distributions independently across genes, marginalize continuous parameters, and finally infer the species tree jointly. This approach can still be called co-estimation because we combine results from multiple genes and *adjust* their distribution jointly at the end. This insight is not new for species tree inference (Ané *et al.*, 2007; Larget *et al.*, 2010, pioneered the idea in the method BUCKy) or improving gene trees (Szöllõsi *et al.*, 2013). Finally, building on the success of quartet-based methods for handling ILS (e.g. Chifman and Kubatko, 2014; Mirarab *et al.*, 2014a), we estimate quartet species trees using a likelihood-based approach but combine the quartet species trees heuristically using supertree methods. Lest the reader worries about lowered taxon sampling and increased long branch attraction (LBA) when using quartets, we note that gene tree estimation is performed on the full set of taxa, but the amalgamation step uses *induced* quartets.

Based on these insights, we introduce a method called quartet-based co-estimation (QuCo for short) that takes as input a Bayesian posterior tree distribution per each of *k* genes, infers the distribution of quartet trees in that input, and summarizes the posteriors in a 3×k table per quartet. Next, for each quartet of species, it computes the maximum likelihood species tree topology and its single internal branch length in the coalescent unit, marginalizing over gene trees. It then improves the gene tree topologies using the species tree. Finally, it combines the inferred quartet species trees to obtain a final tree topology on the complete set of taxa (Supplementary Fig. S1). We evaluate the method on a set of simulations with four to 101 species and a real bee dataset and show that it increases accuracy while providing a path for scalable co-estimation.

## 2 Materials and methods

### 2.1 QuCo: maximum likelihood quartet species trees

We start with the maximum likelihood quartet species tree inference. Throughout, we assume gene trees are made of single-copy orthologous genes and differ due to ILS only, as modeled by MSC. The input to QuCo is the posterior distributions of the gene trees. It computes the maximum likelihood species tree for each quartet of species. Then, using that estimated species tree, it updates the gene tree posterior distributions. While our method for analyzing each quartet is based on likelihood calculations with several simplifying assumptions, to extend to more than four species, we rely on the heuristic method of examining all or a subset of quartets, a procedure we introduce at the end.

#### 2.1.1 Marginalized likelihood of the quartet species trees

For a quartet of species {A,B,C,D}, we denote the three topologies AB|CD, AD|BC, AC|BD by j∈{1,2,3}. Let $D=\{D_{1},\ldots ,D_{k}\}$ be the set of sequences obtained for each available gene. Given D, we seek to compute the likelihood of the species tree, parameterized by θ=(t,d) where t∈{1,2,3} is the topology and *d* is the internal branch length in coalescent unit. This parameterization fully identifies the distribution of unrooted gene tree *topologies* (Allman *et al.*, 2011). We seek the species tree likelihood marginalized over all possibilities for the *k* gene trees, and show the log-likelihood function as l(θ;D)=log(P(D;θ)). Let $C={\left\{1,2,3\right\}}^{k}$. Any set of *k* quartet tree topologies, one per gene, can be indexed by a tuple $c=(c_{1},\ldots ,c_{k})\in C$. Let the true gene tree *topologies* be represented by $G^{*}=(G_{1}^{*},\ldots ,G_{k}^{*})$. Then, our goal is to maximize:

(1) $$\begin{array}{c} P(D;\theta )=\sum_{c\in C}P(D|G^{*}=c;\theta ).P(G^{*}=c;\theta )\\ =\sum_{c\in C}\overset{\text{sequence likelihood}}{\overset{︷}{P(D|G^{*}=c;\theta )}}.\prod_{i=1}^{k}\overset{\text{gene tree likelihood}}{\overset{︷}{P(G_{i}^{*}=c_{i};\theta )}} \end{array},$$

where the last equation uses the conditional independence of gene trees for a fixed species tree. Working on quartet gene tree topologies makes the calculation of gene tree likelihood trivial. Under the MSC model (Allman *et al.*, 2011; Pamilo and Nei, 1988), for any j∈{1,2,3}:

(2) $$P(G_{i}^{*}=j;\theta =(t,d))=\{\begin{array}{cc} 1-2/3e^{-d} & \;\text{if}\;j=t\\ 1/3e^{-d} & \;o.w \end{array}\;.$$

However, working on gene tree topologies (*c*) makes sequence likelihood calculation challenging because we cannot readily write it as a product over genes. To do so, we need all continuous parameters (gene tree substitution unit branch lengths and rate matrices), which we jointly specify using *r_i_* for each gene and $r=(r_{1},\ldots ,r_{k})$. Letting *f*(*r*) be the density function,

(3) $$\begin{array}{c} P(D|G^{*}=c;\theta )=\int_{r}f(r;\theta )P(D|r,G^{*}=c;\theta )dr\\ =\int_{r}\prod_{i=1}^{k}f(r_{i};\theta )P(D_{i}|r_{i},G_{i}^{*}=c_{i})dr\\ =\int_{r}\prod_{i=1}^{k}f(r_{i};\theta )\frac{P(r_{i},G_{i}^{*}=c_{i}|D_{i})P(D_{i})}{f(G_{i}^{*}=c_{i},r_{i})}dr \end{array},$$

where the second equation uses the fact that given all gene tree parameters, gene sequence data are independent of each other and the species tree, and given the species tree, gene trees (thus *r_i_*) are independent.

*Assumptions.* Even for a quartet, computing (3) is not easy. To move forward, we make two assumptions regarding branch lengths. (i) We assume $f(G_{i}^{*}=c_{i},r_{i})=\frac{1}{3}f(r_{i})$, which is reasonable by symmetry when the species tree is *not* given. It requires assuming that *a priori* all three unrooted gene tree topologies are equiprobable, sequence evolution parameters are independent from gene tree topology, and substitution unit branch lengths are independent from *unrooted* gene tree topologies. (ii) We assume $f(r_{i};\theta )=f(r_{i})$. The species tree clearly impacts the distribution of coalescent unit gene tree branch lengths. Typical ways of mapping branch lengths to substitution units assume distributions over population size and mutation rates. These two parameters are ideally drawn per branch, or else gene trees will be ultrametric. When drawn per branch, substitution unit branch lengths are still dependent on the species tree, though the dependence reduces as the variation of rates across branches increase. We assume an extreme case where the mutation rate branch lengths are drawn from distributions independent from the species tree parameter θ. In other words, each branch of the gene tree is assigned a substitution unit length that is independent of the coalescent units length of internal branch (*d*). We also assume that other continuous parameters (e.g. rate matrices) are either constant across the tree or drawn from distributions independent from θ. These assumptions are not entirely realistic but have the advantage of allowing arbitrary and unlimited deviations from the clock, eliminating the need to assume any clock models. Also, they make (3) tractable. Let **P** be the 3×k matrix where $P_{j,i}=P(G_{i}^{*}=j|D_{i})$. Then:

$$\begin{array}{c} P(D|G^{*}=c;\theta )=\int_{r}\prod_{i=1}^{k}f(r_{i})\frac{P(r_{i},G_{i}^{*}=c_{i}|D_{i})P(D_{i})}{\frac{1}{3}f(r_{i})}dr=\\ A\int_{r}\prod_{1=1}^{k}P(r_{i},G_{i}^{*}=c_{i}|D_{i})dr=\\ A\prod_{i=1}^{k}\int_{r_{i}}P(r_{i},G_{i}^{*}=c_{i}|D_{i})dr_{i}=\\ A\prod_{i=1}^{k}P(G_{i}^{*}=c_{i}|D_{i})=A\prod_{i=1}^{k}P_{c_{i},i} \end{array}$$

where $A=\prod_{1}^{k}3P(D_{i})$, and integral and product swap in the third line is possible because no term has two elements of *r*. Replacing RHS in (1):

(4) $$P(D;\theta )=\sum_{c\in C}A\prod_{i=1}^{k}P_{c_{i},i}P(G_{i}^{*}=c_{i};\theta )=A\prod_{i=1}^{k}\sum_{j=1}^{3}P_{j,i}.P(G_{i}^{*}=j;\theta ),$$

where the second equation uses the fact that for any 3×k matrix ${\left(x\right)}_{j,i}$, we have $\sum_{c\in C}\prod_{i=1}^{k}x_{c_{i},i}=\prod_{i=1}^{k}\sum_{j=1}^{3}x_{j,i}$ (easy to confirm).

To compute matrix **P** (posterior gene tree topology probabilities marginalized over branch lengths and substitution parameters), many options are available (Fourment *et al.*, 2020). We mainly take advantage of Bayesian MCMC sampling implemented in standard methods such as MrBayes (Ronquist *et al.*, 2012). Thus, the input to QuCo is a set of *k* gene tree posterior distributions, each inferred separately on its full set of taxa without a species tree. The fraction of times any tree topology appears in the MCMC chain (after some burnout period) is a valid approximation of its posterior probability, marginalized over branch length and other continuous parameters, giving us all values of **P**. We also approximate **P** using normalized quartet log-likelihood as implemented in IQ-TREE (-wql) (Minh *et al.*, 2020). Either way, recalling (2), note that:

$$\begin{array}{c} \sum_{j=1}^{3}P_{j,i}.P(G_{i}^{*}=j;\theta )=(1-2/3e^{-d})P_{t,i}+1/3e^{-d}(1-P_{t,i})\\ =P_{t,i}+e^{-d}(1/3-P_{t,i}) \end{array}$$

which, when replaced in (4), gives us the log-likelihood function:

(5) $$\begin{array}{c} l(\theta =(t,d);P)=\text{log}(A)+\text{log}(\prod_{i=1}^{k}(P_{t,i}+e^{-d}(1/3-P_{t,i})))\\ =A\prime +\sum_{i=1}^{k}\;\text{log}\;(P_{t,i}+e^{-d}(1/3-P_{t,i})), \end{array}$$

where A′ is independent of θ and thus can be ignored. Note that the likelihood is a function of both the topology *t* and the branch length *d*.

For each t∈{1,2,3}, we compute $l\prime (t)=\text{arg}{\text{max}}_{d}l((t,d);P)$ numerically; then, we simply select the topology *t* with the maximum l′(t) value as the species tree. Maximizing the l(θ;P) function numerically is easy because it is twice differentiable and while it is not a convex function of *d* (the sign of its second derivative changes with different input parameters), we can prove (see Appendix A.1):

Proposition 1. *For a fixed* $\tilde{t}$*, the* $l((\tilde{t},d);P)$  *function (5) can have only one maximizer for* 0<d<∞.

Thus, we can seek the global maximum of l((t,d);P) for each t∈{1,2,3} by simple numerical search using any modern optimizer package. We use scipy.optimize package with the constraint *d *>* *0 imposed using the trust-region constrained algorithm (Conn *et al.*, 2000). To help faster convergence, we provide the first and second derivatives of l(.) to the optimizer, as shown in Appendix A.1.1. Finally, we add a small pseudo-count of $10^{-8}$ to every element of **P** and normalize it appropriately.

#### 2.1.2 Gene tree updates

Once a species tree θ=(t,d) is inferred, QuCo updates the gene tree posterior distribution to:

(6) $$P(G_{i}^{*}=j|D_{i};\theta )=\frac{P_{j,i}.P(G_{i}^{*}=j;\theta )}{\sum_{a=1}^{3}P_{a,i}.P(G_{i}^{*}=a;\theta )},$$

where $P(G_{i}^{*}=a;\theta )$ is computed using (2). This update is what makes the method a co-estimation. Note that this approach is not an iterative method switching between updating gene tree topologies and re-estimating species trees; if attempted, the gene tree updates can only increase the probability of the selected species tree compared to the alternative topologies, and will not lead to a change in the next iteration.

#### 2.1.3 More than four species

To move beyond four species, QuCo uses a heuristic supertree approach that ignores the dependency between quartets and analyzes them independently. We first select a set of quartets such that the resolution of all these quartets (perhaps in addition to auxiliary information such as a guide tree) is sufficient to infer the species tree. The simplest choice is to select all $\left(\begin{array}{c} n\\ 4 \end{array}\right)$ quartets but we describe an alternative below. Once the set of quartets is selected, QuCo induces all trees in the MCMC samples of all *k* gene trees down to each selected quartet to compute the quartet posterior probabilities. Thus, for each quartet, a 3×k matrix is obtained. Note that this step, while conceptually simple, needs to process a very large number of trees and thus needs to be implemented with care to obtain high efficiency. Next, for each quartet, we infer the maximum likelihood species tree as described earlier, obtaining a set of quartet species trees. The last step is to combine all the quartet species trees into a full tree using a quartet amalgamation method. While any such method can be used for this step, we will use ASTRAL (as a supertree method, not as a gene summary method) and will show that using wMaxCut (Avni *et al.*, 2015) generates very similar results.

*Sampling quartets*. For sufficiently small datasets (e.g. <50 species), we afford to examine all the quartets. For larger input, we use a two-step approach. We first run ASTRAL on input gene trees, defined for each gene as the majority-rule consensus (MRC) of the trees in the input distribution for that gene. Next, we contract all the branches in the ASTRAL tree with local-pp support (Sayyari and Mirarab, 2016b) less than a threshold (default: 1.0). We then use an algorithm to sample quartets around polytomies of the resulting multifurcating guide tree, and this strategy focuses the quartet sampling on difficult parts of the tree. For a multifucating node *u* of degree *d*, we sample a single species from each side of *u* (or a uniformly sampled set of 12 sides when *d *>* *12), and choose all quartets made with sampled species. To choose a species on each side, we use probabilistic sampling: Rooting the tree at *u*, we traverse the tree to reach a leaf, at each node choosing a child uniformly at random. The closer the leaf is to the polytomy, the higher the chance we sample it. We repeat the sampling procedure many times, and by default, reduce the rounds proportionally to the degree *d* (default number of rounds: 1200/d). Note that since each round generates $\left(\begin{array}{c} d\\ 4 \end{array}\right)$ quartets for d≤12, we perform fewer rounds for larger *d*. In the end, in addition to the QuCo-resolved species tree quartets, we give the multifurcating guide tree to the subsequent supertree method (e.g. ASTRAL) as input. Thus, in effect, we use QuCo to resolve polytomies of the input guide tree.

### 2.2 Simulations

#### 2.2.1 New simulation datasets and protocols

**Fig. 1.**
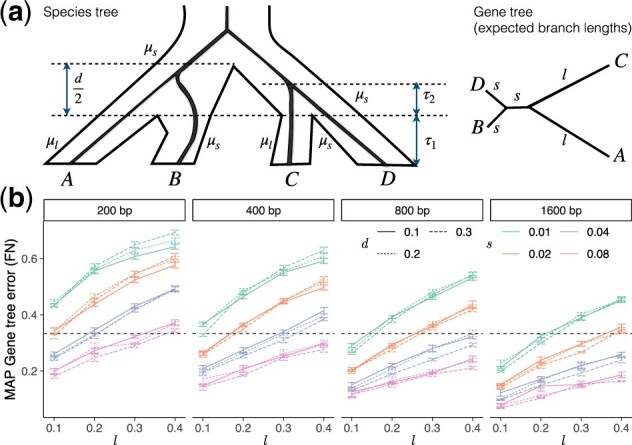
Felsenstein’s zone simulation. (**a**) Each gene tree branch length is scaled by μ_*s*_ and/or μ_*l*_; for example, the length of the terminal branch of *C* becomes $\mu_{l}\tau_{1}+\mu_{s}\tau_{2}$. Rates μ_*s*_ and μ_*l*_ are selected such that terminal branches of A and C in the unrooted gene tree have expected length *l*, and other branches have expected length *s*, as shown. (**b**) MAP gene trees estimated using MrBayes with simulations in Felsenstein’s zone can have large estimation error, especially when l/s is high and sequence lengths (boxes) are short

*Felsenstein’s zone*. Long branch attraction is among the most challenging sources of systematic bias in phylogenomics (Brinkmann *et al.*, 2005; Jeffroy *et al.*, 2006), and Roch *et al.* (2019) have shown that both summary methods and concatenation are inconsistent under conditions that induce LBA. Thus, we perform simulation studies close to the Felsenstein zone (Felsenstein, 1978) to assess the resiliency of our method to LBA. To do so, we designed a way of simulating gene trees that tend to be in Felsenstein’s zone. First, gene trees in coalescent units are generated according to MSC on a fixed balanced species quartet tree (Fig. 1a). Each branch of the species tree has one of two mutation rates μ_*s*_ and μ_*l*_ assigned to it. Each gene tree branch length is multiplied by the rates of corresponding species branches (a gene tree branch may cover one to three species tree branches) to obtain their length in substitution units. We set μ_*s*_ and μ_*l*_ so that two non-sister terminal branches (B and D) and the internal branch in the unrooted gene trees share a short expected length *s* and the other two terminal branch lengths have expected length *l*. Setting μ_*s*_ and μ_*l*_ properly requires a lemma (proved in Appendix A.2):

Lemma 2. *Under MSC, for a balanced quartet species tree with internal branch lengths* $\frac{d}{2}$  *(Fig. 1a), the expected length of terminal branch lengths in unrooted gene trees above the speciation nodes is* $\tau_{2}=1-\frac{1}{3}e^{-d}$.

Let τ_1_ be the fixed coalescent unit terminal branch lengths for all species, and let μ_*l*_ and μ_*s*_ be mutation rates assigned to the tree as shown in Figure 1. The expected substitution unit length of terminal branches of A and C (*l*) and terminal branches of B and D (*s*) are: $l=\mu_{l}\tau_{1}+\mu_{s}\tau_{2}$ and $s=\mu_{s}(\tau_{1}+\tau_{2})$. Thus, we assign $\mu_{s}=\frac{s}{\tau_{1}+\tau_{2}}$ and $\mu_{l}=\frac{l-\tau_{2}s/(\tau_{1}+\tau_{2})}{\tau_{1}}$ so that the expected branch lengths are as desired. Finally, note that the expected length of the internal unrooted gene tree branch is 1 in coalescent units and μ_*s*_ in substitution units. To force the expected internal branch length in substitution units to be also *s* (as in Felsenstein’s zone), we need to set $\tau_{1}=1-\tau_{2}=\frac{e^{-d}}{3}$.

With this setting, each simulation is parameterized by the coalescent unit internal branch *d* (controlling amount of ILS) and expected length of long and short terminal branches, *l*, *s*, respectively. LBA is expected for high l/s. We used this simulator to create very hard conditions meant to break methods. We vary *l*, *s*, and *d* in 48 combinations, each with 20 replicate runs. We set d∈{0.1,0.2,0.3}, which corresponds to 40%, 45%, and 51% of gene trees matching the species tree. We use the Dendropy package (Sukumaran and Holder, 2010) to simulate 500 gene trees (simulated trees are called *true* gene trees hereafter) under neutral coalescent model conditioned on a species tree shown in Figure 1. For each *d*, we consider 16 combinations of short and long branch lengths: s∈{0.01,0.02,0.04,0.08} and l∈{0.1,0.2,0.3,0.4} and convert gene tree branch lengths to substitution units, as described earlier. Then, we use INDELible (Fletcher and Yang, 2009) to simulate sequences down these trees, setting the sequence length to 200, 400, 800, and 1600 bp. Thus, in total, we have 48 × 4 = 192 model conditions, 3840 replicates, and 1 920 000 gene trees. We infer gene trees using MrBayes and ensure convergence by checking the average SDs of split frequencies, which is less than 0.08 for all runs with 99 percentile equal to 0.025.

We compare QuCo to ASTRAL and BUCKy-quartet (Larget *et al.*, 2010). BUCKy has been shown to have accuracy similar to MSC-based co-estimation methods (Chung and Ané, 2011). As input to ASTRAL, we use maximum *a posteriori* (MAP) MrBayes. Gene trees estimated using MrBayes from simulated alignments can have high rates of error, depending on l/s and sequence length (Fig. 1b). Note that a random selection of tree topology will still be correct 1/3 of times; thus, the MAP gene trees have more error (due to LBA bias) than randomly estimated trees in some conditions. Moreover, incorrect gene trees are not randomly distributed but are heavily biased towards putting long terminals (A and C) together. Thus, conditions with gene tree error above 1/3 are particularly difficult. Note that here, the ASTRAL tree is equivalent to the most common topology among the MAP gene trees.

*Anomaly zone*. We simulate a dataset with a 6-taxon caterpillar species tree that based on the calculations presented by Degnan (2013) is in both rooted and unrooted anomaly zone (Supplementary Fig. S2). The anomaly zone refers to species trees that define gene tree distributions where the most likely gene tree is different from the species tree and may present a particularly challenging part of the parameter space (Degnan and Rosenberg, 2006). To make the dataset more realistic, we also create deviations from ultrametricity. We assign substitution unit branch lengths to gene trees by multiplying each coalescent unit length by overall rate 0.02 and a rate multiplier sampled independently from a Gamma distribution with shape and scale set to 5 and 1/5 (mean: 1, variance: 1/5). We generate 10 000 gene trees in total and use INDELible to simulate 600-bp sequences down these trees. We run MrBayes separately on each of these sequences and check convergence by checking the average SDs of split frequencies, which are less than 0.015 for all runs. We divide the 10 000 genes into 50 replicates of 10, 50, 100, and 200 genes, 20 replicates of 500 genes, 10 replicates of 1000 genes, or five replicates of 2000 genes. For each replicate, we run QuCo on all 15 quartets of species and combine them using the exact ASTRAL.

#### 2.2.2 Existing datasets

*30-taxon datasets*. We reuse a dataset simulated by Mai and Mirarab (2017) using Simphy (Mallo *et al.*, 2016) with three model conditions, and 500 genes, each with 50 replicates (sampled out of 100 original replicates). The three conditions are differentiated by their level of deviations from the molecular clock, as controlled by α, which is the inverse of the variance of the rate multipliers applied to gene tree branch lengths. Because of difficulties in running MrBayes to convergence for all of the 3×500×50=75 000 gene trees, we use IQ-TREE instead. We use IQ-TREE -wql option to compute the log-likelihood for all quartet topologies, which we then normalize and exponentiate to approximate posteriors and use as input to QuCo. See Appendix B.1 in Supplementary Material for exact commands. We run QuCo on all $\left(\begin{array}{c} 30\\ 4 \end{array}\right)=27\;405$ quartets and combine these quartet trees using ASTRAL or wMaxCut.

*101-taxon datasets*. We use one model condition of a dataset simulated by us and colleagues (Zhang *et al.*, 2018) with 101 taxa, 400-bp sequences, 200 genes, and 30 replicates sampled out of a total of 50 replicates, each with a distinct species tree. The species trees are simulated under the birth-only process with the birth rate $10^{-7}$, fixed haploid *N*_e_ of 400*K*, and the number of generations sampled from a log-normal distribution with the mean 2.5M. The average normalized RF distance between true species trees and true gene trees was in most replicates in the [0.3, 0.6] range, with an average of 0.46. The simulation process is similar to the 30-taxon dataset and uses Simphy and INDELible. We run two chains of MrBayes MCMC for 600 000 generations on each gene alignment. Here, we use the quartet sampling strategy described before.

## 3 Results

### 3.1 Simulation results

#### 3.1.1 Felsenstein’s zone simulations

**Fig. 2.**
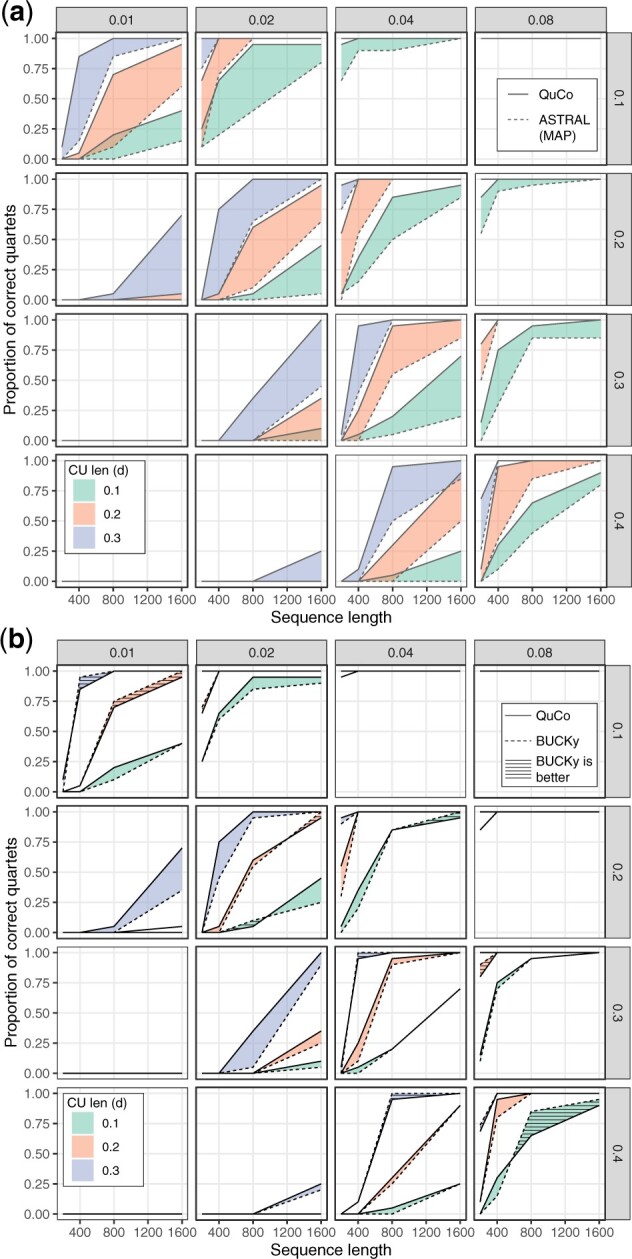
Felsenstein’s zone quartet simulations comparing QuCo to ASTRAL (**a**) or Bucky-Quartet (**b**). Each box shows a combination of long *l* (rows) and short *s* branch lengths (columns), and colors delineate ILS level controlled by *d*. Each ribbon shows the improvement of QuCo over ASTRAL or BUCKy, all run on MrBayes gene trees. When the ribbon is patterned, BUCKy is better than QuCo

*Topological accuracy*. QuCo is at least as accurate as and in many conditions far more accurate than ASTRAL in finding the correct topology (Fig. 2a). Across all conditions, QuCo finds the correct tree in 1953 out of 3840 replicates, whereas ASTRAL is correct in 1572 cases. The improvements are most clear in model conditions where l/s=10. For example, with l=10, s=0.2 and 800-bp sequences, QuCo has 100% and 60% accuracy, respectively, with *d *=* *0.3 and *d *=* *0.2 compared to 65% and 10% for ASTRAL. When *s* and *l* are close, both ASTRAL and QuCo work well. For example, both methods recover the true species tree in all replicates when l/s≤5/2 (top right corner) with *d *=* *0.2 or *d *=* *0.3 and in most cases for *d *=* *0.1. On the other hand, when l/s>20 (bottom left corner), even with 1600-bp sequences, neither method recovers true topology in any replicate; with l/s=20, QuCo recovers the true species tree between 5% and 70% of times if the sequence length is at least 800 bp, but ASTRAL continues to infer the wrong tree in every case.

Compared to BUCKy, QuCo shows improvements in many but not all conditions, and improvements are less substantial (Fig. 2b). When ILS is lower (*d *=* *0.3), the two methods are identical or similar except in three *l*, *s* combinations where QuCo has a substantial advantage for 400 bp or longer alignments and one case where BUCKy has a small advantage with 400-bp alignments. Across all conditions with *d *=* *0.3, QuCo is correct in 811 out of 1280 replicates tested, which is 3% higher than BUCKy (788). With *d *=* *0.2, the two methods are similar to small advantages for QuCo in nine conditions out of 64 and for BUCKy in four conditions. With the highest level of ILS, QuCo and BUCKy are each substantially better in some conditions. Among all species trees tested, the number of times QuCo is correct is 50 times more than BUCKy.

Consistently through all model conditions, longer sequences (hence more signal) in the gene trees result in more accurate species tree estimation, as expected. When sequence are as short as 200 bp, the correct topology is almost never recovered when l/s≥10; with 400-bp alignments, all methods fail in most cases when l/s≥15. Even some difficult cases such as *l *=* *0.3, *s *=* *0.02 or *l *=* *0.4, *s *=* *0.04 are rescued when using QuCo and to a lesser degree using BUCKy as long as sequences are sufficiently long; in these conditions, the accuracy can go from zero up to one with *d *=* *0.3. The impact of longer sequences is also clearly observed in conditions with moderate l/s (e.g. l=5×s=0.1 or l=5×s=0.2) where close to perfect accuracy is obtained by QuCo and BUCKy but not ASTRAL with 1600-bp sequences even with *d *=* *0.1.

As expected, higher levels of ILS (i.e. lower *d*) make inference harder for both methods. There are, however, conditions where QuCo is quite robust to the level of ILS while ASTRAL is not. For example, for *l *=* *0.3, *s *=* *0.04, with 1600-bp sequences, QuCo has 70% accuracy for the highest ILS level and 100% in the other cases. In contrast, ASTRAL accuracy degrades with increased ILS (perfect for *d *=* *0.3, 85% for *d *=* *0.2, and 20% for *d *=* *0.1).

**Fig. 3.**
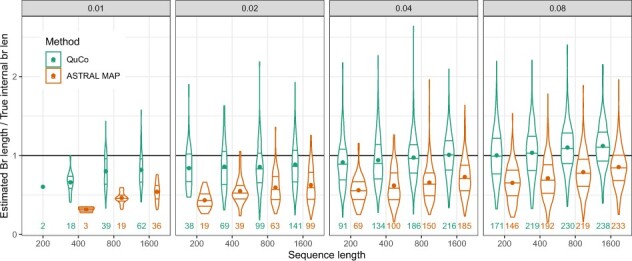
Branch length accuracy on Felsenstein’s zone simulations, showing the distribution of estimated branch length divided by true branch length for correctly estimated species tree (the number of such cases shown in each case). Lines show the four quartiles and the dot shows the mean. Each box corresponds to a value of *s*, combining all *l* values. See Supplementary Figure S5 for better resolution

*Branch lengths*. To evaluate the branch length accuracy, we report the ratio between *d* estimated by QuCo or ASTRAL to the true branch length, only considering cases where the species tree topology is correct. QuCo branch lengths are much closer to true branch lengths than ASTRAL branch lengths with all sequence lengths (Fig. 3). The under-estimation bias of ASTRAL branch lengths as a result of inaccurate gene trees, as shown by Sayyari and Mirarab (2016b), is vastly reduced by QuCo. With the most difficult model conditions, both methods under-estimate the internal branch length while QuCo produces far more accurate estimates. In most model conditions, longer sequences help QuCo to estimate more accurate branch lengths. However, when *s *=* *0.08, QuCo can surprisingly *over*-estimate branch lengths by 12% with ≥800-bp data.

**Fig. 4.**
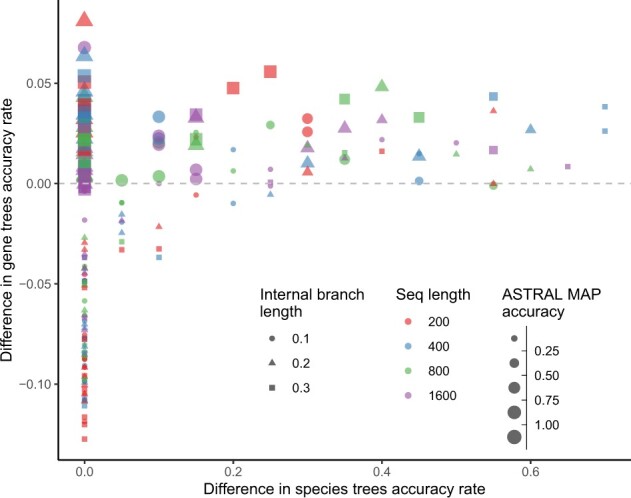
Gene tree estimation error on Felsenstein’s zone simulations. Each dot corresponds to one model condition, with the *x*-axis showing the improvement in species tree accuracy by QuCo compared to ASTRAL and the *y*-axis showing the improvement in the average gene tree accuracy for all genes. The size of dots corresponds to the accuracy of ASTRAL species trees

*Gene tree error*. We evaluate gene tree accuracy by comparing how often the MAP estimate is correct before or after the co-estimation update step performed by QuCo using Equation (6). Unlike universal improvements in the species tree accuracy, the gene tree accuracy of QuCo is mixed (Fig. 4). The quartet gene trees produced by QuCo are better than the original MAP gene trees under most conditions where the species trees are improved compared to ASTRAL and under most ‘easy’ conditions where both ASTRAL and QuCo find the correct tree. However, under the most challenging conditions where neither method can find the correct species tree (e.g. for l/s>20), the QuCo gene trees are *less* accurate than the raw MAP trees. Note that co-estimation by QuCo first computes the species tree and then strictly increases the probability for gene tree topologies that match the species tree at the expense of those that disagree with it. Thus, a reduced gene tree accuracy with incorrect species trees can be expected.

#### 3.1.2 Anomaly zone

**Fig. 5.**
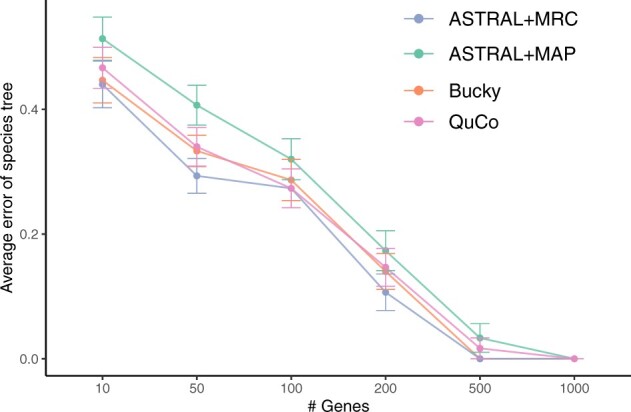
Species tree topological error (mean, standard error) under anomaly zone simulations versus the number of genes

We next test the 6-taxon anomaly zone dataset, where we report the portion of true branches missing in the estimated tree (which is equal to the normalized RF distance because all trees are fully resolved). Here, the error is high for all methods when given tens of genes but decreases quickly when the number of genes increases to hundreds (Fig. 5). As we go to large numbers of genes, we get to zero error with all methods. ASTRAL run on MAP gene trees is slightly less accurate than BUCKy and QuCo, which have similar levels of accuracy. Interestingly, running ASTRAL on MRC gene trees improves its accuracy to levels that match or surpass BUCKy and QuCo.

#### 3.1.3 30-taxon datasets

**Fig. 6.**
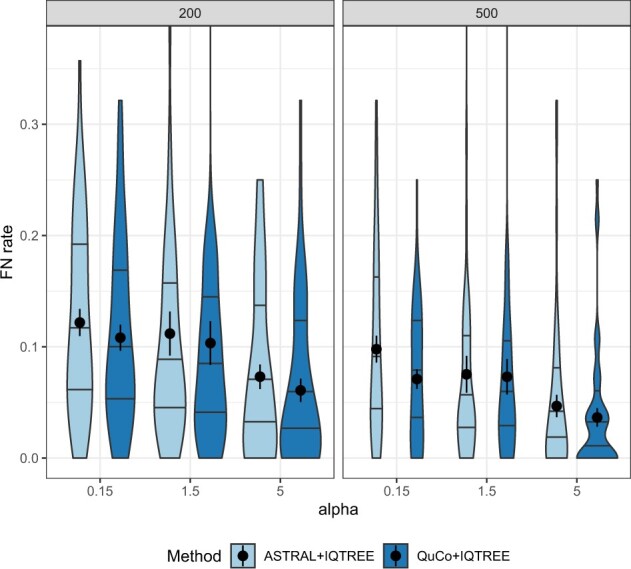
30-taxon dataset. Left: Comparison of the error rate of the species tree generated by running ASTRAL on IQ-Tree ML gene trees and QuCo on IQ-Tree quartet likelihoods with 200 and 500 genes of 30-taxon dataset. The *x*-axis shows deviation from clock represented by parameter α (inverse of the variance of rate multipliers). Each box is over 50 replicates

Here, instead of MrBayes, we run QuCo on maximum likelihood (ML) IQ-TREE gene trees. On the larger 30-taxon dataset, depending on the model condition, QuCo+IQ-TREE either matches or improves on the accuracy of ASTRAL+IQ-TREE (Fig. 6). Note that testing BUCKy was not possible for these larger data. The improvements are obtained both for conditions with high and low deviations from the strict clock but are less clear for conditions with moderate deviations. When deviations are high, accuracy improves as a result of increasing the number of gene trees from 200 to 500 for both methods, but the improvements are larger for QuCo (from mean error of 11% to 7% versus from 12% to 10%). Note that the inputs to ASTRAL and QuCo are not identical in this experiment: The ML gene trees are inferred from the entire set of species, whereas quartet tree likelihoods are inferred per quartet. Thus, it is reasonable to expect the input to QuCo to be more prone to LBA than ASTRAL, making it more remarkable that it has a lower error in its output.

On this dataset, we also compare ASTRAL and wMaxCut as supertree methods for the step where quartet species trees are combined (Supplementary Fig. S3). The two ways of combining the quartets are similar to ASTRAL performing slightly better (worse) for low (high) deviations from the clock. We use ASTRAL as the default supertree method elsewhere.

#### 3.1.4 The large 101-taxon dataset

On the larger dataset, where the sampling strategy is necessary, the number of internal nodes in the ASTRAL guide trees ranges from 69 to 84 (mean 78) compared to 99 nodes for a fully resolved tree. Our sampling strategy selects between 3127 and 51 272 quartets (mean: 22 508), which we resolve using QuCo. The polytomies range from small (degree 4) to a maximum degree of 19 (mean: 5), and the guide trees have no incorrect branches. After the polytomies of the guide tree are refined using QuCo, we observe a 12% decrease in the average topological error (Supplementary Table S1) compared to the original ASTRAL tree run on MrBayes MRC gene trees and a 40% decrease compared to ASTRAL run on Fasttree gene trees with branches with bootstrap support below 10% contracted, which is our recommended setting from Zhang *et al.* (2018). Note that here MAP becomes impossible to estimate; thus, we use the MRC summary instead.

### 3.2 Application on a biological dataset

We test QuCo on the dataset of *Pseudapis* genus of bees of Bossert *et al.* (2021) with 32 species and 1291 UCEs from the subfamily *Nomiinae* (Halictidae). We use the MrBayes posterior estimations from the original study and run QuCo on all 35 960 quartets. Then, we combine quartets using ASTRAL and enrich its search space with 853 IQ-TREE gene trees.

The species tree that we recover by running QuCo matches the ASTRAL tree reported by Bossert *et al.* (2021) on gene trees created using Phylobayes, which is designed to reduce LBA (Lartillot *et al.*, 2007). Bossert *et al.* (2021) have reported 30 ASTRAL trees from gene trees created by ML and Bayesian methods, and these trees differ in five areas compared to the concatenation tree. The tree reported by QuCo differs from concatenation in two nodes and is identical for the other nodes (Supplementary Fig. S4) and also differs from ASTRAL on MrBayes in one of those two nodes. These two nodes involve the two samples with the worst sequencing success, *Ruginomia rugiventris* and *Stictonomia schubotzi*. Both of these taxa have over 75% undetermined positions in the concatenated matrix and are present in less than half of all loci, making them hard to place.

## 4 Discussion

We introduced the algorithm QuCo for quartet co-estimation of species trees and gene trees. We showed that QuCo had better accuracy than ASTRAL in quartet simulations with LBA. By considering gene tree uncertainty, QuCo also outperformed ASTRAL under the anomaly zone simulations when the number of genes was limited. It can be easily proved that if all $P_{t,i}$ values are either 0 or 1 (i.e. in the absence of gene tree estimation uncertainty), QuCo is equivalent to picking the most frequent quartet as the species tree, as is done in ASTRAL. The improvements, then, are a result of considering gene tree uncertainty. As the number of genes increased, better handling of uncertainty appeared to be less consequential as QuCo and ASTRAL converged in accuracy in the anomaly zone simulations. Compared to the alternative co-estimation method BUCKy, QuCo had a small advantage in accuracy; however, note that BUCKy has limited scalability (Yang and Warnow, 2011).

Despite the fact that the method first infers gene trees independently, it is a co-estimation method because the species tree maximizes the joint likelihood marginalized over all possible gene tree topologies. This marginalization was computationally tractable because we consider quartets independently and can use a simple Equation (2) for the likelihood. The likelihood of gene trees for more taxa is much harder to compute and requires exponential time (Wu, 2012). Alternatively, one can assign branch lengths to gene trees in the same unit as the species tree to make likelihood calculation fast. However, this imposes a different challenge: the need to assume a distribution for mutation rates and population sizes, which further increases the number of parameters that need to be sampled. Many co-estimation methods side-step this challenge by assuming a strict molecular clock, an assumption that decades of research has proved problematic.

By focusing on gene tree topologies and species tree branch length in the coalescent unit, we avoid using a strict gene tree clock model while making the problem *easier* to solve (i.e. requiring fewer parameters). Our approach did require assuming the independence of substitution branch lengths from the species tree topology and internal branch length. This assumption, which is probably violated on all biological datasets, can be understood as ignoring the shared information about branch lengths across genes. Thus, it makes the co-estimation less powerful than a method that does consider correlation between branch lengths, especially if it can do so under a correct model of rate evolution. However, we note that none of our experimental tests made any such assumptions. Thus, the high empirical performance of the method indicates these assumptions are not lethal; they just may reduce the power of the method in exchange for scalability. Furthermore, the caveat in working with topologies is that QuCo does not output gene tree branch lengths. Moreover, using topologies makes the likelihood calculations fast for four species, and going beyond four species requires the heuristic supertree approach. Thus, our simplifying assumptions have the benefits of: (i) freeing us from assuming restrictive models of rate change across the tree, and (ii) fast calculations of likelihood; however, they also render our method more heuristic than full Bayesian co-estimation methods.

Interestingly, while QuCo clearly increased species tree accuracy, it appeared less effective in increasing gene tree accuracy, especially when the species tree was not improved. This trend is in contrast to some of the existing co-estimation methods, such as the iterative method of Wang and Nakhleh (2018), that are effective in increasing the gene tree accuracy but less so in terms of the species tree. While these patterns call for further study in the future, two points should be emphasized. By marginalizing over gene tree distribution, QuCo can improve the species tree, even when the maximum likelihood gene tree (given the species tree) is not improved. Moreover, when QuCo fails to improve the quartet species tree, it has no chance of improving the gene tree, and in fact, it likely degrades it. Finally, note that QuCo essentially generates a distribution over topologies for each quartet in each gene tree. When more than four species are available, a quartet amalgamation method such as wMaxCut needs to be used to compute the final updated gene trees.

The scalability of QuCo comes from the fact that the inference for each quartet is fast. Given the **P** matrix, the optimization step takes a fraction of a second per quartet. Even on the 30-taxon data, the optimization step takes close to 1 h across all 27 405 quartets. Given MrBayes outputs, computing **P** is conceptually easy, and with appropriate implementation, can be fast (with I/O being the bottleneck). The entire running time, including the I/O heavy calculation of **P**, is still reasonably fast. For example, for the biological dataset with 32 species, QuCo took 12 h to analyze all 35 960 quartets across 1200 genes with no parallelization (mostly calculation of **P**). This can be run in parallel; using 80 cores, 13 min is enough to analyze all quartets. The final step of combining the quartet trees is also fast, taking 36 s using ASTRAL and only a couple of seconds using wMaxCut. The more time-consuming part of the pipeline, by far, is to run MrBayes on all gene trees. However, this step can be done in parallel and is much more manageable than co-estimation. For example, Bossert *et al.* (2021) reported that each MrBayes run on 32 taxa took 6.7 min on average. Running methods like MrBayes on thousands of genes with hundreds of species is doable. For even larger datasets where MrBayes may not scale, our results showed that using IQ-TREE quartet likelihoods, which are extremely fast to compute, can be very accurate. To summarize, 101-taxon is by no means the limit of the method.

Analyzing a large number of taxa (e.g. beyond 50) requires quartet selection strategies instead of using all $\left(\begin{array}{c} n\\ 4 \end{array}\right)$ quartets. Quartet subsampling is a problem that has been studied in the literature (Davidson *et al.*, 2018; Snir *et al.*, 2008) and solutions with quadratic (Sayyari and Mirarab, 2016a) or even quasi-linear (Brown and Truszkowski, 2011) numbers of quartets have been proposed. We left the exploration of such approaches to the future. Instead, we tried a simple method where a guide tree (here, ASTRAL) is estimated and uncertain branches are contracted. The polytomies left in the tree are the difficult parts of the tree, hence our desire to focus the quartet sampling around the polytomies. Our probabilistic leaf sampling strategy uses the well-established insight that short quartets (those with leaves closer to the polytomy) are easier to resolve correctly than long quartets (Erdos *et al.*, 1999; Snir *et al.*, 2008). While our sampling strategy proved effective, we believe better methods may be possible, including those that would guarantee that the number of quartets increases quasi-linearly or quadratically with the number of species.

## Data availability

The data underlying this article are available in GitLab at https://gitlab.com/mrabiee/quo-data.

## Funding

This work has been supported by the NSF (IIS-1845967) to S.M. and M.R. Computations were performed through XSEDE allocations, which is supported by the NSF (ACI-1053575).

*Conflict of Interest*: none declared.

## Supplementary Material

btac265_Supplementary_Data

## Appendix

### A.1 Likelihood maximization

Proof of Proposition 1. We can rewrite (5) without the logarithm as

$$P(D;\theta )=A\prod_{i=1}^{k}\left(\beta_{i}+x\alpha_{i}\right)$$

where $\beta_{i}=P_{t,i}$, and $\alpha_{i}=1/3-P_{t,i}=1/3-\beta_{i}$, and $x=e^{-d}$. Since α_*i*_s and β_*i*_s are constant, P(D;θ) is a polynomial in *x* and can have at most *k* roots of the form

$$-\frac{\beta_{i}}{\alpha_{i}}=\frac{\beta_{i}}{\beta_{i}-1/3}$$

and *k*−1 local optima that must each be between two roots. Note that only valid values of *x* in our optimization are 0<x<1 corresponding to 0<d<∞. Thus, we are interested in local optima in that region. However, every root of the form shown is negative when β<1/3 and is >1 for $1/3<\beta_{i}\leq 1$. Thus, none the roots are in the 0<x<1 region we are interested in. Since the polynomial has no root in 0 to 1, it can have only one local optimum in that region. Note also that x→0 and x→1 both result in non-negative likelihood values, and thus, there must be one valid maximizer to the function.

#### A.1.1 Derivatives

The derivatives of the log likelihood function are given to the optimizer:

$$\begin{array}{c} l\prime (t,d;P)=\sum_{i=1}^{k}\frac{3P_{t,i}-1}{1+3P_{t,i}(e^{d}-1)})\\ l\prime \prime (t,d;P)=\sum_{i=1}^{k}\frac{3P_{t,i}e^{d}(1-3P_{t,i})}{{\left(1+3P_{t,i}(e^{d}-1)\right)}^{2}} \end{array}$$

### A.2 Simulating long branch attraction with MSC

Proof (sketch), Lemma 2. Recall the balanced quartet tree with length $\frac{d}{2}$ above the two speciation nodes as shown in Figure 1. By symmetry, all terminal branches have the same length in coalescent units. W.l.o.g., we take branch *A*. Recall that the probability density function for the coalescence of two lineages in time *t* before present is given by $e^{-t}$ in coalescent time units. We compute the expectation by conditioning on three scenarios: (*I*) lineages *A* and *B* coalesce before the root, (*II*) lineage *A* and *B* do not coalesce before the root but lineage *C* and *D* do, and (*III*) neither lineage *A* and *B* nor lineages *C* and *D* coalesce before the root. Let *c*_3_ and *c*_4_ be the expected time to coalescence between *A* and another branch among three and four branches in total, respectively. Then, the expected length of terminal branch of *A* above its common ancestor with *B* in the species tree is:

$$\overset{I}{\overset{︷}{\int_{0}^{\frac{d}{2}}te^{-t}dt}}+\overset{\text{II}}{\overset{︷}{e^{-\frac{d}{2}}(1-e^{-\frac{d}{2}})\left(c_{3}+\frac{d}{2}\right)}}+\overset{\text{III}}{\overset{︷}{e^{-2\frac{d}{2}}\left(c_{4}+\frac{d}{2}\right)}}$$

The first term simply follows from the definition of expectation. The second and third terms first compute the probability of lack of coalescence ($e^{-d/2}$) on one or both sides, and multiple by the expected length in each case. The *c*_3_ and *c*_4_ terms give the expected length in the root (by definition) and d/2 is added to account for the length on the branch above common ancestor of *A* and *B*. We compute *c*_3_ again using conditional expectation:

$$c_{3}=\frac{1}{3}\frac{2}{3}+\frac{1}{3}\left(\frac{1}{3}+1+1\right)=1$$

The first term conditions on *A* being the first lineage to coalesce with another (probability $\frac{2}{3}$), and uses the fact that the expected length of the first coalescent among *N* lineages is $1/\left(\begin{array}{c} N\\ 2 \end{array}\right)=\frac{1}{3}$. The second term is conditioned on *A* not being the first lineage to coalesce with another and is computed similarly. In this scenario, *A* will continue for the first coalescent event (length$\frac{1}{3}$), up to the final coalescence $1/\left(\begin{array}{c} 2\\ 2 \end{array}\right)=1$; we also need to add the length of the branch to the other side of the deepest coalescence because we are dealing with unrooted trees. With similar logic, we can compute:

$$c_{4}=\frac{1}{6}\frac{1}{2}+\frac{1}{3}\left(\frac{1}{6}+\frac{1}{3}\right)+\frac{1}{6}\left(\frac{1}{6}+\frac{1}{3}+1+1\right)=\frac{2}{3}$$

where the three terms correspond to *A* being the first, the second, and the last branch to coalesce. Replacing these terms in the first equation, we get the expectation equals:

$$1-e^{-\frac{d}{2}}\left(1+\frac{d}{2}\right)+e^{-\frac{d}{2}}(1-e^{-\frac{d}{2}})\left(1+\frac{d}{2}\right)+e^{-2\frac{d}{2}}\left(\frac{2}{3}+\frac{d}{2}\right)=1-\frac{e^{-d}}{3}$$
