# Supplementary material for: QuCo: quartet-based co-estimation of species trees and gene trees
Source: Bioinformatics. 2022 Jun 27;38(Suppl 1):i413–21. doi: 10.1093/bioinformatics/btac265 (PMC9235488; doi:10.1093/bioinformatics/btac265)
Supplement: btac265_Supplementary_Data [file btac265_supplementary_data.zip › btac265-Suppl_data/S5.pdf]

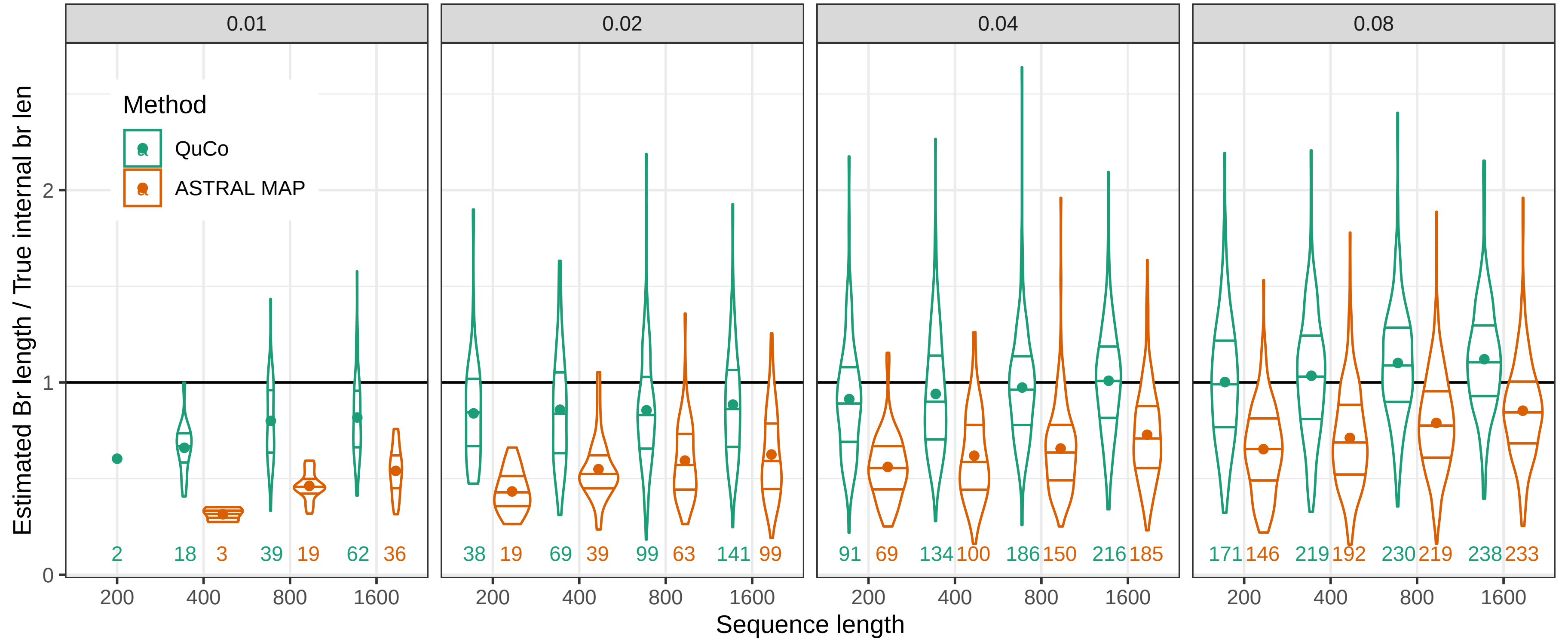
0.01
0.02
0.04
0.08
Method
QuCo
ASTRAL MAP
Estimated Br length / True internal br len
0
1
2
2
18
3
39
19
62
36
38
19
69
39
99
63
141
99
91
69
134
100
186
150
216
185
171
146
219
192
230
219
238
233
200
400
800
1600
Sequence length
