# Supplementary material for: QuCo: quartet-based co-estimation of species trees and gene trees
Source: Bioinformatics. 2022 Jun 27;38(Suppl 1):i413–21. doi: 10.1093/bioinformatics/btac265 (PMC9235488; doi:10.1093/bioinformatics/btac265)
Supplement: btac265_Supplementary_Data [file btac265_supplementary_data.zip › btac265-Suppl_data/Supplement_193[AU].pdf]

B Supplementary Material

B.1 Commands

B.1.1 IQ-Tree

iqtree -s seqfile -st DNA -lmap ALL -wql -seed 1234567890 -m GTR -pre iqtree -n 0 -redo -keep-ident

B.1.2 MrBayes

begin mrbayes; set autoclose=yes nowarn=yes ;execute seq.nex; lset nst=6 rates=gamma; mcmc nruns=2 mcmcdiagn=yes samplefreq=500 stoprule=yes stopval=0.015 file=seq.nex; sumt; end;

picture(0,0)(-35,0)(1,0)30 (0,35)(0,-1)30 picture

picture(0,0)(35,0)(-1,0)30 (0,35)(0,-1)30 picture

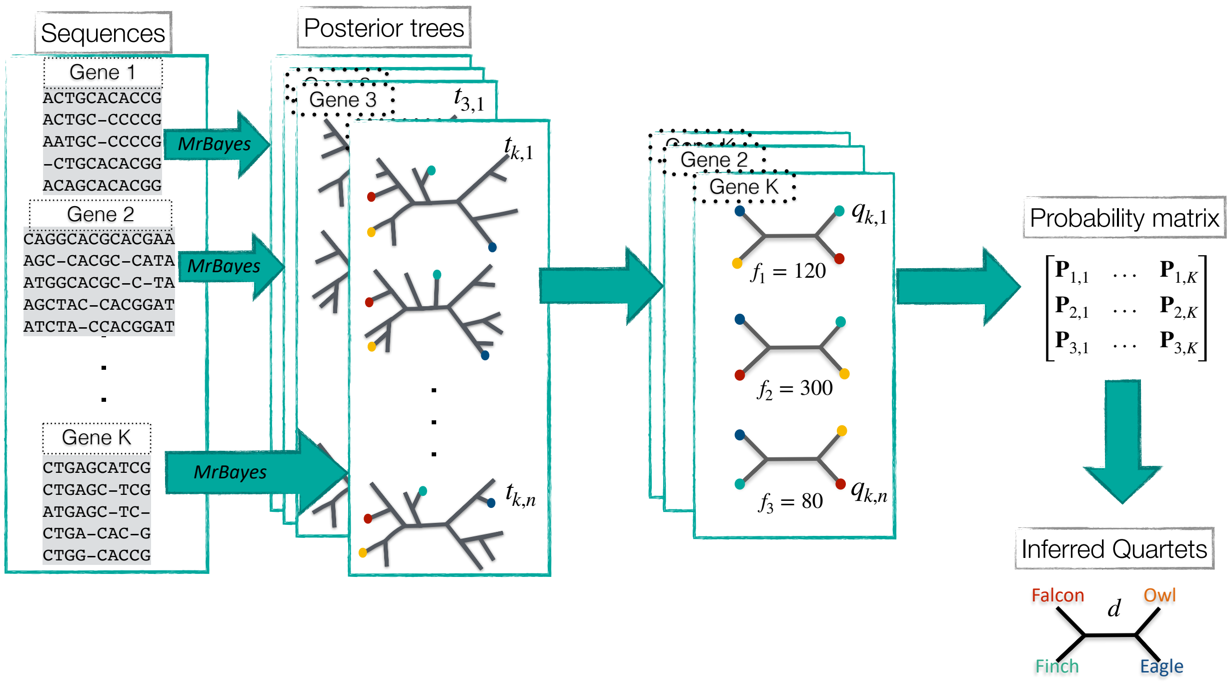

Fig. S1. Pipeline for inferring quartet topology and branch length from sequences of more than four species. Colored dots show a sampled quartet.

picture(0,0)(-35,0)(1,0)30 (0,-35)(0,1)30 picture

picture(0,0)(35,0)(-1,0)30 (0,-35)(0,1)30 picture

"output" — 2022/3/24 — page 11 — #11

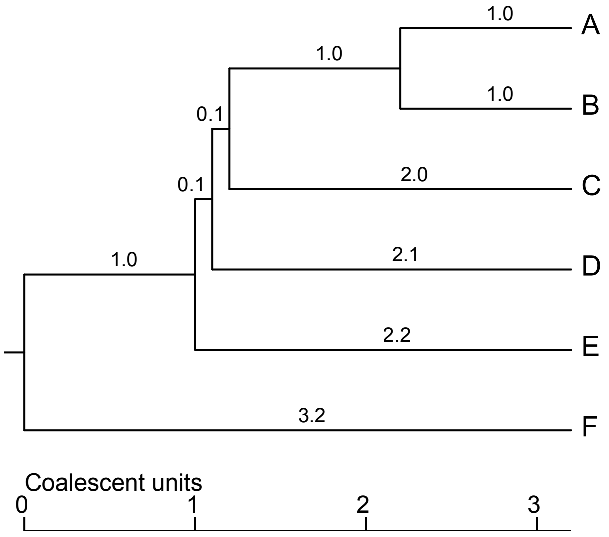

Fig. S2. True species tree used in anomaly zone analyses. According to results by Degnan (2013), this tree is in unrooted and rooted anomaly zones due to the presence of two short branches surrounded by long branches.

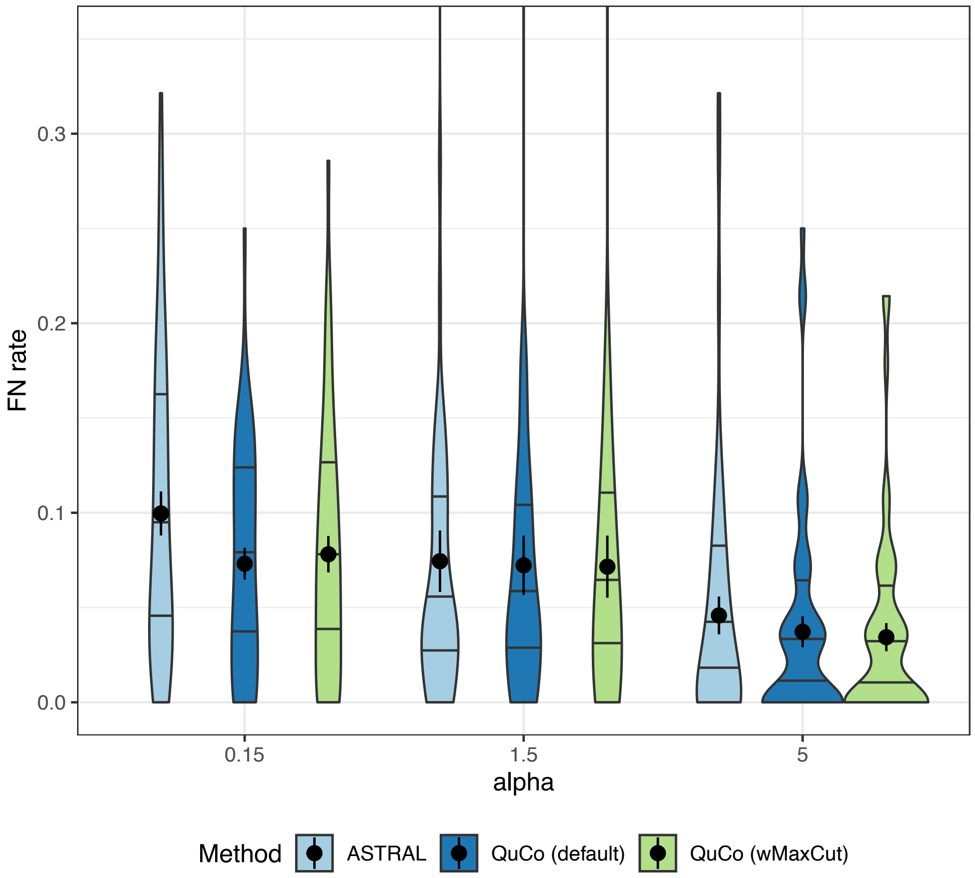

Fig. S3. Comparison of the error rate of the species tree generated by running two versions of QuCo: the default version using ASTRAL for combing quartets, and the version combining quartets using wMaxCut. All methods are compared on 50 replicates with 500 genes.

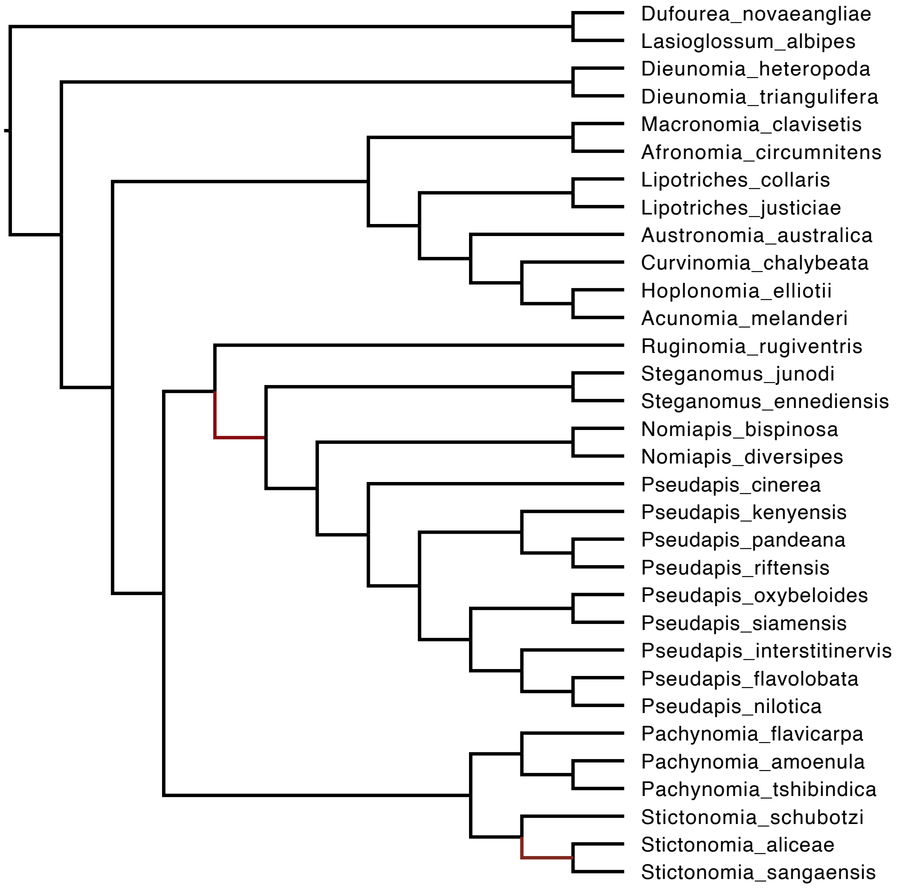

Fig. S4. Species tree created by running ASTRAL on all quartets estimated by QuCo on bees dataset

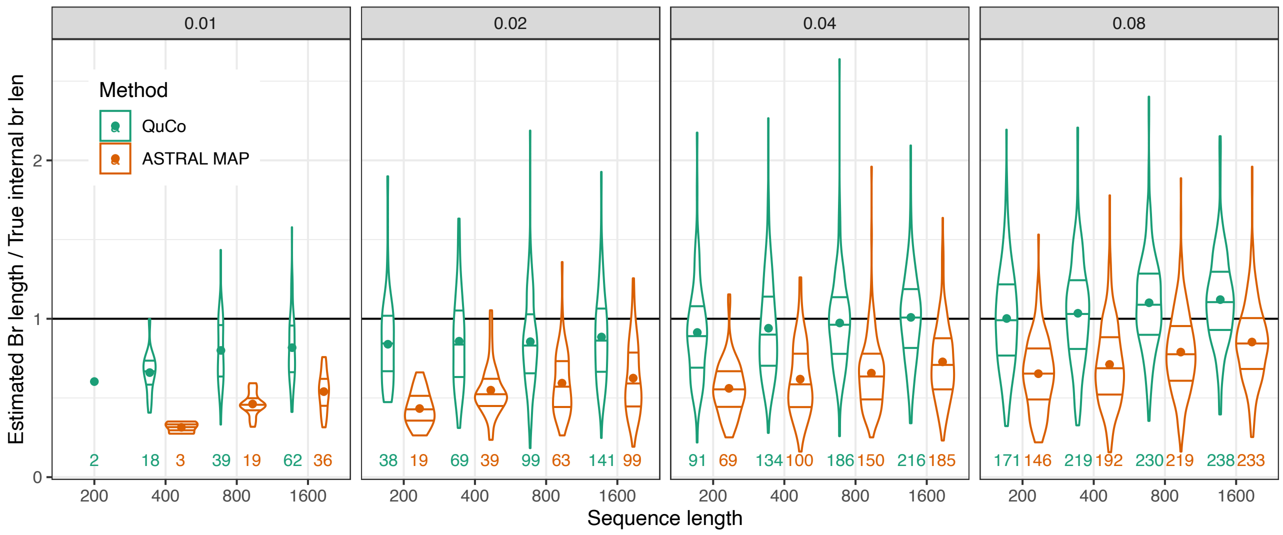

Fig. S5. Branch length accuracy on Felsenstein's zone simulations, showing the distribution of estimated branch length divided by true branch length for correctly estimated species tree (the number of such cases shown in each case). Lines show the four quartiles and the dot shows the mean. Each box corresponds to a value of s, combining all l values.

Table S1. QuCo results with sampling on ASTRAL-III dataset of 101 species compared to ASTRAL run on MRC trees of MrBayes and Fasttree gene trees

	QuCo	ASTRAL+MRC	ASTRAL+Fasttree (10%)
Mean	4.6%	5.2%	7.7%
Median	41.%	5.1%	7.1%
