# Supplementary figures and images for: QuCo: quartet-based co-estimation of species trees and gene trees
Source: Bioinformatics. 2022 Jun 27;38(Suppl 1):i413–21. doi: 10.1093/bioinformatics/btac265 (PMC9235488; doi:10.1093/bioinformatics/btac265)

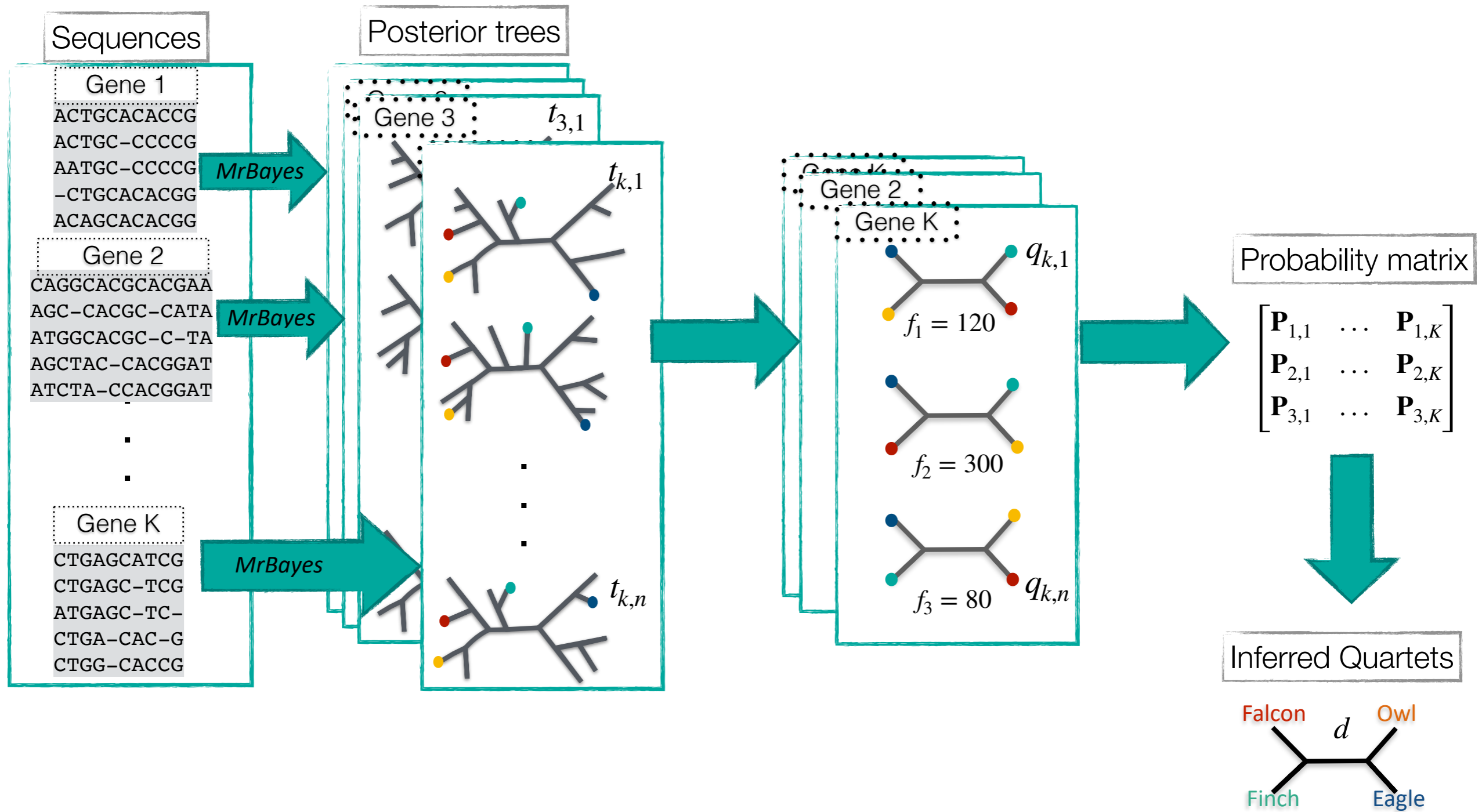

Supplement: btac265_Supplementary_Data [file btac265_supplementary_data.zip › btac265-Suppl_data/S1.pdf]

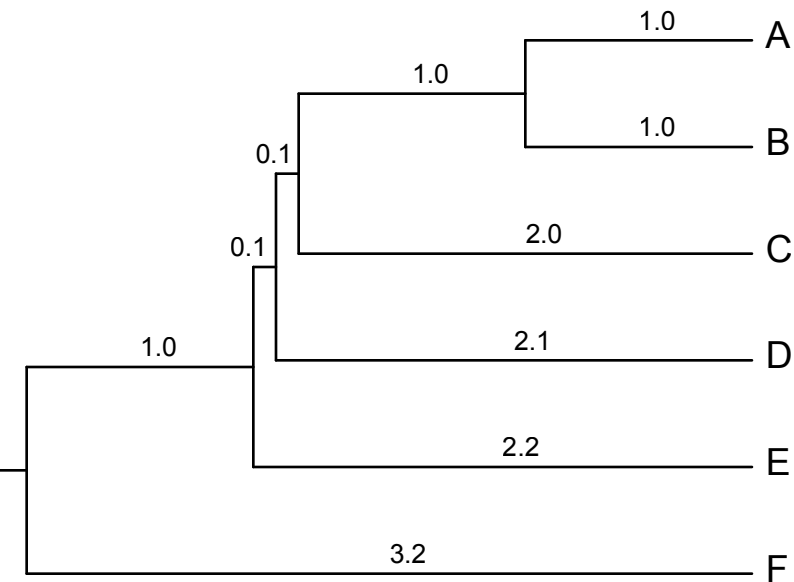

Coalescent units

0

1

2

3

Supplement: btac265_Supplementary_Data [file btac265_supplementary_data.zip › btac265-Suppl_data/S2.pdf]

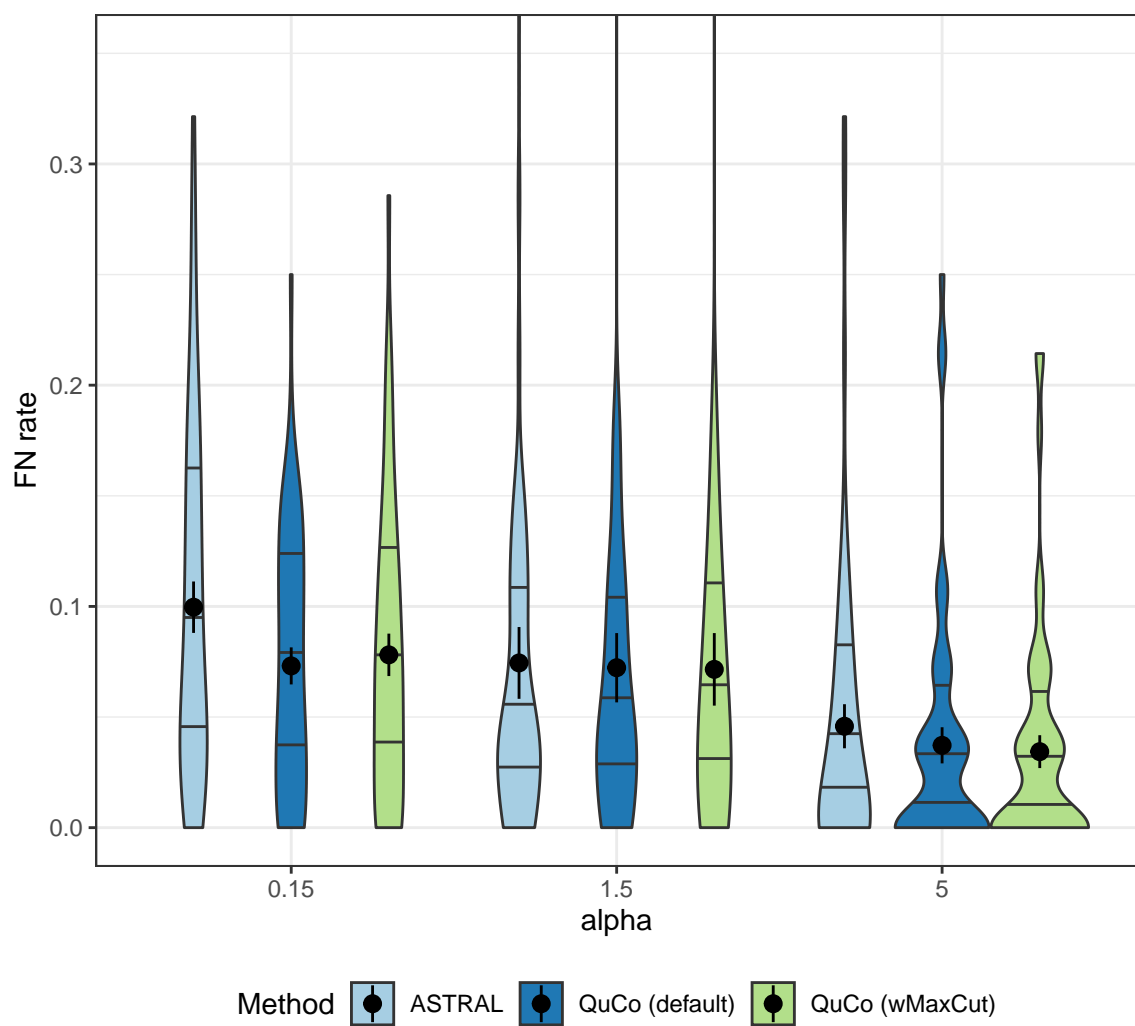

Supplement: btac265_Supplementary_Data [file btac265_supplementary_data.zip › btac265-Suppl_data/S3.pdf]

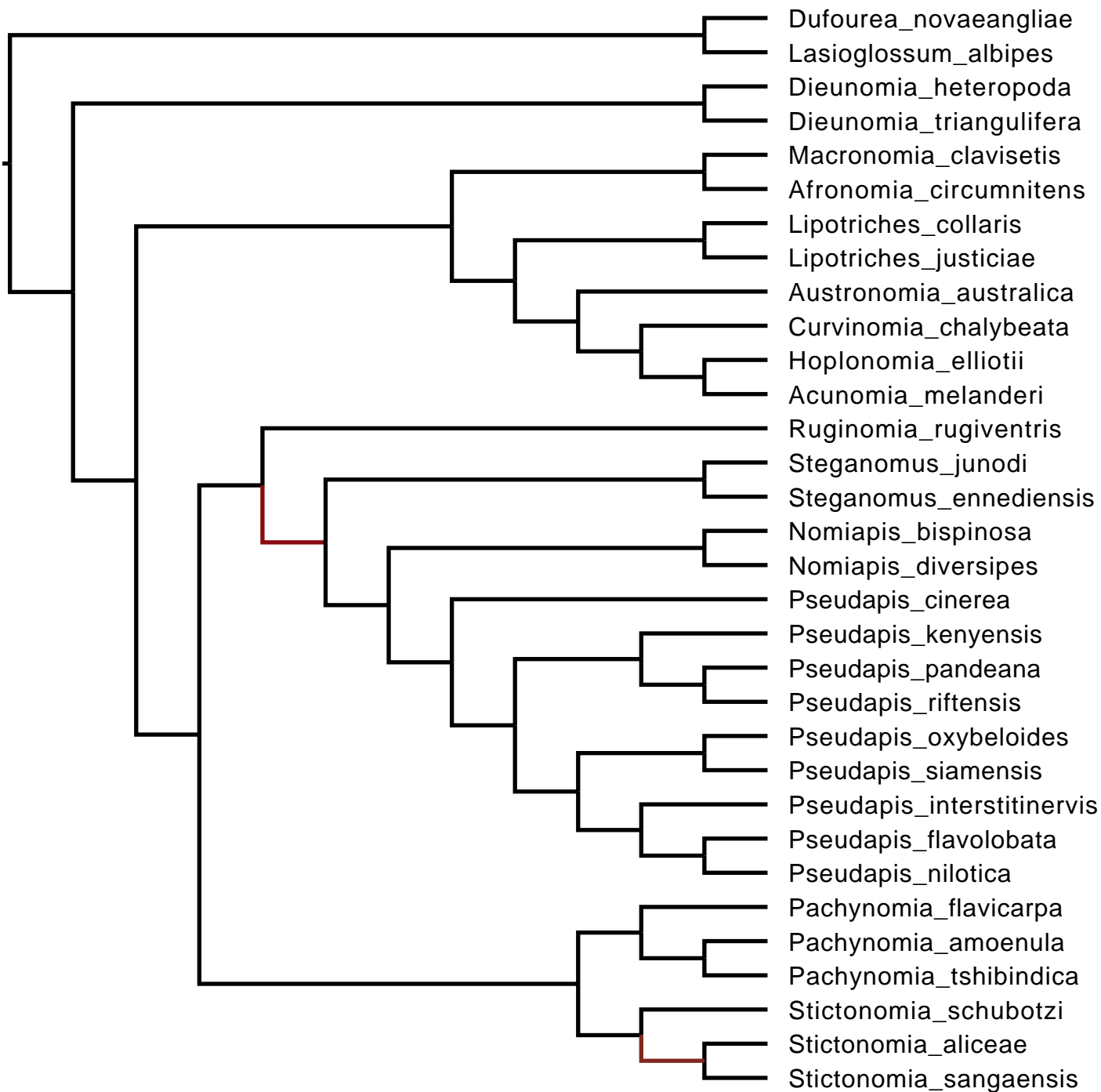

Supplement: btac265_Supplementary_Data [file btac265_supplementary_data.zip › btac265-Suppl_data/S4.pdf]
